# Longitudinal shear wave elasticity measurements of millimeter-sized biomaterials using a single-element transducer platform

**DOI:** 10.1371/journal.pone.0266235

**Published:** 2022-04-06

**Authors:** Shao-Lun Lu, Pei-Yu Chao, Wei-Wen Liu, Kun Han, Jason Chia-Hsien Cheng, Pai-Chi Li

**Affiliations:** 1 Graduate Institute of Biomedical Electronics and Bioinformatics, National Taiwan University, Taipei, Taiwan; 2 Division of Radiation Oncology, National Taiwan University Hospital, Taipei, Taiwan; 3 Graduate Institute of Oncology, National Taiwan University College of Medicine, Taipei, Taiwan; 4 Department of Electrical Engineering, National Taiwan University, Taipei, Taiwan; University of Montreal, CANADA

## Abstract

Temporal variations of the extracellular matrix (ECM) stiffness profoundly impact cellular behaviors, possibly more significantly than the influence of static stiffness. Three-dimensional (3D) cell cultures with tunable matrix stiffness have been utilized to characterize the mechanobiological interactions of elasticity-mediated cellular behaviors. Conventional studies usually perform static interrogations of elasticity at micro-scale resolution. While such studies are essential for investigations of cellular mechanotransduction, few tools are available for depicting the temporal dynamics of the stiffness of the cellular environment, especially for optically turbid millimeter-sized biomaterials. We present a single-element transducer shear wave (SW) elasticity imaging system that is applied to a millimeter-sized, ECM-based cell-laden hydrogel. The single-element ultrasound transducer is used both to generate SWs and to detect their arrival times after being reflected from the side boundaries of the sample. The sample’s shear wave speed (SWS) is calculated by applying a time-of-flight algorithm to the reflected SWs. We use this noninvasive and technically straightforward approach to demonstrate that exposing 3D cancer cell cultures to X-ray irradiation induces a temporal change in the SWS. The proposed platform is appropriate for investigating *in vitro* how a group of cells remodels their surrounding matrix and how changes to their mechanical properties could affect the embedded cells in optically turbid millimeter-sized biomaterials.

## Introduction

The mechanical properties of cell microenvironments constantly change as the resident cells synthesize, degrade, and remodel the extracellular matrix (ECM) to promote specific cellular activities such as differentiation and migration [1]. ECM stiffening resulting from excessive matrix deposition and cross-linking has been recognized as a characteristic mechanical and physical feature of solid tumors [2]. This composition change produces a temporal variation of ECM elasticity, which greatly affects tumor proliferation, metabolism, invasion, and metastasis [3]. In addition to cancer biology, the findings of seminal studies of tissue engineering have also suggested roles of the scaffold stiffness in regenerative medicine. From adhesion to differentiation, appropriate cellular behaviors from early embryogenesis onward are affected by tissue stiffness [4,5].

A three-dimensional (3D) culture with tunable matrix stiffness is needed to characterize the mechanobiological interactions of elasticity-mediated cellular behaviors during cancer progression and cell development. Most conventional studies have used ECM-coupled polyacrylamide gels of defined stiffness [6–9], with the investigators modulating the stiffness of the hydrogel at the beginning of each observation. There are usually no further elasticity measurements and no information on ECM remodeling obtained after the cells are encapsulated into the matrix. However, the mechanical interactions between cells and the ECM are dynamic, and so an imaging system capable of providing elasticity measurements on a millimeter-scale sample with an appropriate temporal resolution is desirable for monitoring the matrix’s mechanical dynamics. The development of such platforms requires consideration of the dimensions of the sample, imaging accessibility, biological relevance, technical difficulty, and cost for elasticity measurements that best suit a particular 3D cell culture.

Bulk elasticity measurements made using rheometry with direct contact and stress induction would result in sample destruction, and therefore are not suitable for repeated interrogations. On the other hand, atomic force microscopy (AFM) has been widely adopted in biomechanical studies to measure the elasticity of 3D cell cultures [10]. An AFM device is equipped with a micro- or nanosized tip that measures the sample resistance to an indentation made on a very small area of the substrate. While AFM offers high spatial resolution on the micrometer scale, it is inherently a surface or subsurface compliance measurement involving pinpoint probing. The sample surface is interrogated by grid scanning, which restricts its applicability to the typical dimensions of a 3D culture. The time-consuming AFM measurements (typically taking 2–3 hours to produce a 150 μm × 150 μm stiffness map) also have adverse impacts on samples encapsulating live cells [10]. Finally, AFM remains an invasive approach that produces mechanical destruction [11], making it more appropriate for endpoint measurements.

Temporal measurements of the elasticity in a 3D culture or an engineering tissue reveal dynamic cell–ECM interactions. They are usually best performed using noncontact imaging-based methods, including Brillouin microscopy [11,12], magnetic resonance elastography (MRE), and ultrasound shear wave (SW) elastography [13–15]. Confocal Brillouin microscopy probes the sample’s bulk modulus by detecting frequency shifts of the transmitted laser light caused by thermally generated acoustic phonons. Despite providing a 3D elasticity map with ultrahigh spatial resolution, Brillouin spectroscopy is fundamentally based on light scattering, and so the sample opacity restricts the penetration of the probing laser and hence its application to depths within hundreds of micrometers of the sample surface. MRE have been developed to quantify viscoelastic properties of tissues, including breast, brain, and liver[16]. MRE measures shear wave propagation, which is often initiated by external excitation or tissue palpation. It can provide millimeters to a sub-millimeter resolution of stiffness [17]. While MRE has been widely used for clinical application or tissue-level stiffness measurement, it is less frequently adopted for subcentimeter-sized biomaterials due to the high experimental cost and the difficulty in coupling a high-frequency actuator to a small and stiff sample [18].

Shear wave (SW) imaging is a quantitative elasticity imaging technique in which a time-varying force is generated either externally by an external vibrator [19] or internally by a focused acoustic radiation force (ARF) [20] to produce ensuing mechanical waves. Compared to applying the force at the surface, ARF is a body force acting throughout the volume of the applied acoustic beam, with the strongest SW propagating away from the pushing-beam’s axis at the depth of the acoustic focus [21]. It is essentially instantaneously to provide the relationship between displacement and elasticity and is weakly influenced by the surface boundaries. Most importantly, ARF with coupling medium could rely on no physical contract with the sample; Therefore, when probing fragile 3D hydrogels or engineering biomaterials containing living cells for nondestructive longitudinal studies, ARF is the frequently chosen approach. Several other methods generate the sample perturbations, including pneumatic [22,23] and optical excitations [24]. The former does not require direct contact, while the tubing and mechanics to deliver the air pulse limit its miniaturization for small samples. Due to a relatively low-frequency response and bandwidth, the air-pulse approach is also more susceptible to boundary conditions [25]. Optical excitation usually uses pulsed laser irradiation, which is converted into localized heat expansion of the tissues. Though offering a focused excitation, laser-induced thermal damage on collagenous material has been reported [26].

Ultrasound-based and optical-based shear wave elasticity imaging (SWEI) has been applied to monitor temporal variations in 3D collagen-based cell culture elasticity. Compared to the acoustic method, the optical approach generally exhibits improved spatial resolution and accuracy of elasticity imaging. It could also be performed without exogenous scatters cell-laden biomaterials. We have previously characterized the temporal and spatial dynamics of the ECM stiffness in a millimeter-sized 3D cell culture using a laser speckle contrast shear wave imaging (LSCI-SW) system [27]. We used an ultrasound transducer to generate an SW in the culture sample, which was illuminated using a coherent light source. The propagation speed of the SW was determined by analyzing the laser-speckle patterns. While that platform allows the imaging system to perform noninvasive, noncontact elasticity measurements on the cell culture system, the requirement for optical transparency restricts its application to biological tissues due to their typical intrinsic scattering properties. Moreover, a system based on both ultrasound and optical speckle imaging principles requires a high level of operator expertise for platform setup and accurate measurements.

On the other hand, conventional ultrasound-based SWEI is appropriate for biological tissues, with substantial volumes and intrinsic optical scattering. By adopting a high-frequency transducer, the spatial resolution of the submillimeter-level could also be achieved. The platform will not need to couple the optical and ultrasonic setups if using ARF to generate shear waves. However, ultrasound-based SWEI, especially with an ARF-induced approach, usually requires a relatively large sample volumes to prevent resonant waves [28]. Therefore, it has not been widely adopted in investigations of cell-matrix interactions on small biomaterials, despite of the extensively use in clinical research or animal models. For an optically turbid sample, Kuo *et al*. demonstrated temporal variations in the elasticity of a millimeter-sized, 3D collagen-based cell culture using ultrasound-based high-frequency SWEI) [29]. Despite revealing the spatiotemporal dynamics of the elasticity of the ECM, the system utilizes two ultrasound transducers that need to be carefully aligned to perform mechanical scanning to achieve confocal shear wave imaging. The bulky system setup also requires the cell sample to be more than 5 cm in diameter, which makes it unsuitable for nonvascularized cell cultures. Moreover, the size of the culture hinders sample section for use in further biological studies and increases the costs of investigations.

Discoveries emerging from tuning temporal changes in forces in the 3D environments of biomimetic materials have demonstrated the profound effects of temporal variations of stiffness on cellular behaviors, which are possibly more significant than the influence of static stiffness [4,30]. Characterization of the dynamics of elastic moduli of the cellular environment is becoming as much as necessary for depicting the static stiffness of biological samples. Here we present an ultrasound-based SWEI platform of a single transducer that allows longitudinal observations of the dynamics of the matrix stiffness of a millimeter-sized 3D culture system, which is also suitable for optically turbid biomaterials.

## Methods and materials

### Shear wave elasticity measurements

**Fig 1:**
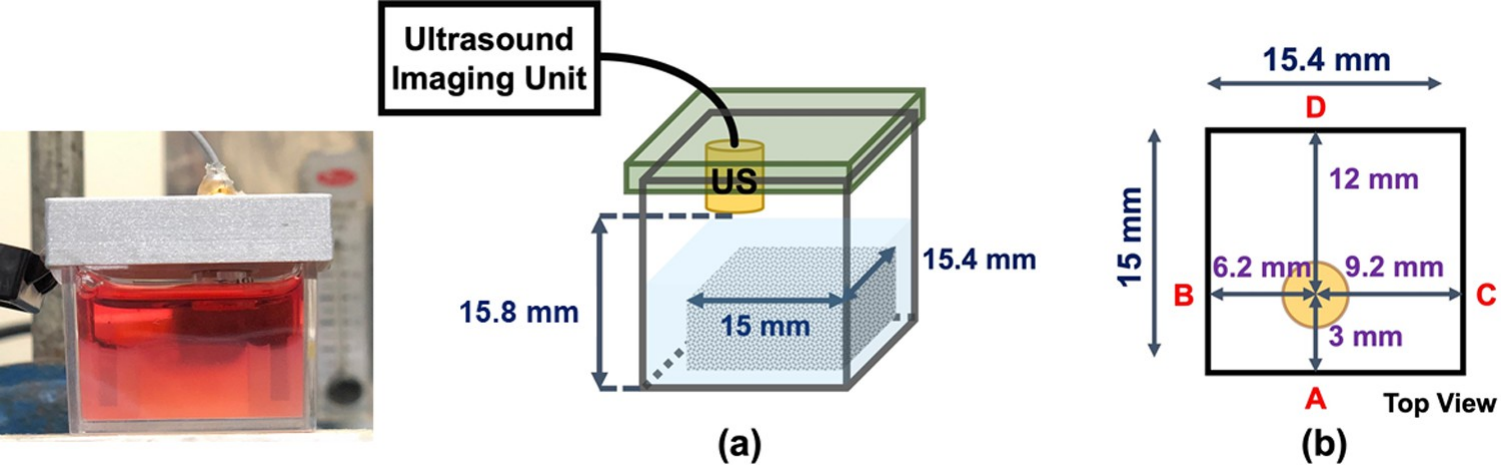
(a) Schematic of the single-element transducer ultrasound shear wave elasticity measurement system. (b) illustration of the position of the ultrasound transducer (US) relative to the side boundaries of the sample.

A custom-made single-element 20 MHz focused ultrasound transducer with an *f*-number of 2 and a focal length of 14 mm was securely installed on a designed fixture mounted onto the sample container. The ultrasound transducer face was approximately 15.8 mm from the bottom of the sample container. Fig 1(A) shows a schematic of the elasticity measurement system, and Fig 1(B) illustrates the position of the ultrasound transducer relative to the side boundaries of the sample.

The single-element focused ultrasound transducer performs both SW generation and detection. The ultrasound transducer was firstly driven by a 2000-cycle sinusoidal pulse (peak-to-peak voltage of 140V) to generate ARF in the sample. The drive voltage and pulse duration (0.1 ms) were to those been used in the previous research [31]. The ARF could successfully produce the ensuing shear waves in an agarose gel, inside which the propagation of shear wavefronts was imaged through a full-field laser speckle contrast imaging approach. The ultrasound transducer was then switched to pulse-echo mode to acquire 400 A-lines at a sampling frequency of 5 kHz. The ultrasound imaging unit (Prospect, S-Sharp, Taipei, Taiwan) generates the driving signal to the ultrasound transducer and acquires the subsequent SW images. A one-dimensional autocorrelation algorithm was applied to the demodulated data to obtain the axial displacements induced by the propagation of the SWs.

The transducer fixture was designed so that the one-way distances between the focal point of the ultrasound transducer and the side boundaries were fixed at 3.0, 6.2, 9.2, and 12.0 mm. Each SW propagates transversely through the sample and is reflected from its four side boundaries, with the reflected SWs arriving at the focal point of the ultrasound transducer separated by approximately 6 mm. With known propagation distances, the shear wave speed (SWS) of the sample can be thus obtained by averaging the two-way propagation distances for the arrival times of the four SWs; that is, $SWS=\frac{\sum_{i}^{4}\frac{d_{i}}{t_{i}}}{4}$.

### Sample preparation

**Fig 2:**
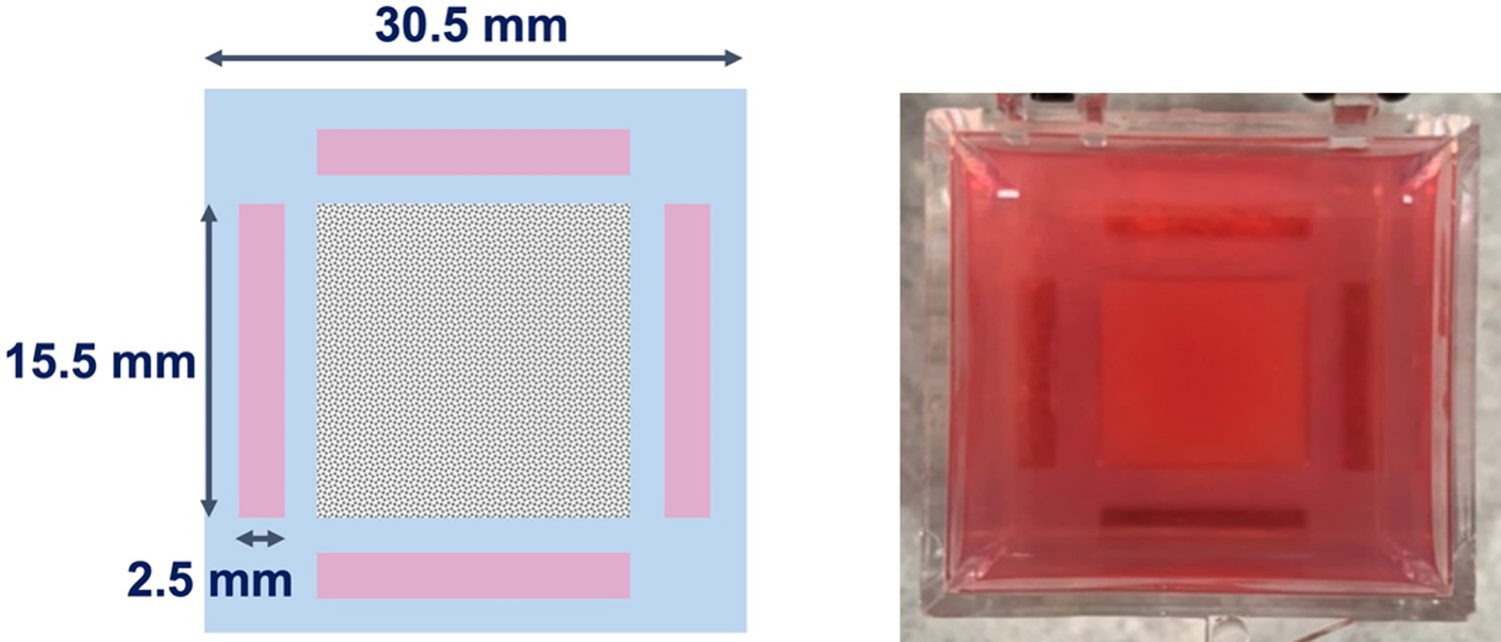
Top view of the sample and the agarose supporting structure (blue) along with side channels (pink) reserved for cell culture experiments.

As illustrated in Fig 2, an approximately cubic sample with dimensions of 15 mm ⨉ 15.4 mm (width ⨉ length) was surrounded by an agarose structure made with 0.5% agarose. The agarose structure supports the sample during polymerization. It acts as a buffer that resulted in the smallest one-way distance between the side boundary of the sample to the focal point of the ultrasound transducer being 3 mm.

Different types of homogeneous phantoms were made using 0.3% and 0.4% agarose (Lonza, Basel, Switzerland), as well as 3.5% and 5% gelatin (Sigma-Aldrich, Missouri, United States). Silicon dioxide (S5631, Sigma-Aldrich) was added at a concentration of 0.16% to act as scatterers to produce the necessary speckle pattern for measuring the SWS. The heights of the phantoms were purposely made to be different. For each of the phantoms made for the single-element transducer ultrasound SW measurement system, a separate sample (with width ⨉ length ⨉ height dimensions of 9 mm ⨉ 12 mm ⨉ 18 mm) with identical ingredients was constructed, and this was imaged using an LSCI-SW system for validating the shear modulus of the sample [27].

### Cancer-cell-laden gel

The matrix gel sample comprised a mixture of Matrigel (Corning, New York, United States) and collagen type I (Corning) in the cell culture experiments. Three sets of cell cultures with different concentrations of Matrigel matrix and collagen type I were used: 5 mg/ml Matrigel with 1, 2, and 3 mg/ml collagen type I. The matrix gel was prepared by first mixing collagen type I with a pH neutralizing buffer containing 1 M NaOH, PBS, 0.9 mM CaCl_2_, 0.5 mM MgCl_2_, and sterilized distilled water. The human hepatocarcinoma cell line Huh7 (JCRB cell bank, Okayama, Japan) was trypsinized from a culture plate and resuspended at 3.4x10^6^ cells/ml in serum-free tumorsphere medium (TSM) before being carefully mixed with Matrigel matrix. The mixture of Matrigel and cells was then added to and thoroughly combined with the collagen mixture. Finally, we added biocompatible silicon dioxide at a mass concentration of 0.18% to the gel mixture as the ultrasound scatterers. The matrix gel was prepared on ice to avoid polymerization. The matrix gel was carefully loaded into the cubic hollow in the agarose structure. The side channels were filled with 200 μl of TSM to provide sufficient moisture to the gel during the polymerization process.

The entire cell culture sample was then placed in an incubator at 37°C for 150 minutes. After polymerization, 7 ml of TSM was added to the top of the culture and the side channels. The medium was replaced every 24 hours to ensure adequate exchanges of nutrients and waste products. After SW measurements, the cell culture sample was fixed in 4% paraformaldehyde and 25% glutaraldehyde (Sigma-Aldrich). Frozen sections with a thickness of 10 μm obtained from the culture sample were stained with H&E and imaged using a bright-field microscope with 10× and 40× objectives (AxioImager M1, Zeiss, Oberkochen, Germany).

## Results

**Fig 3:**
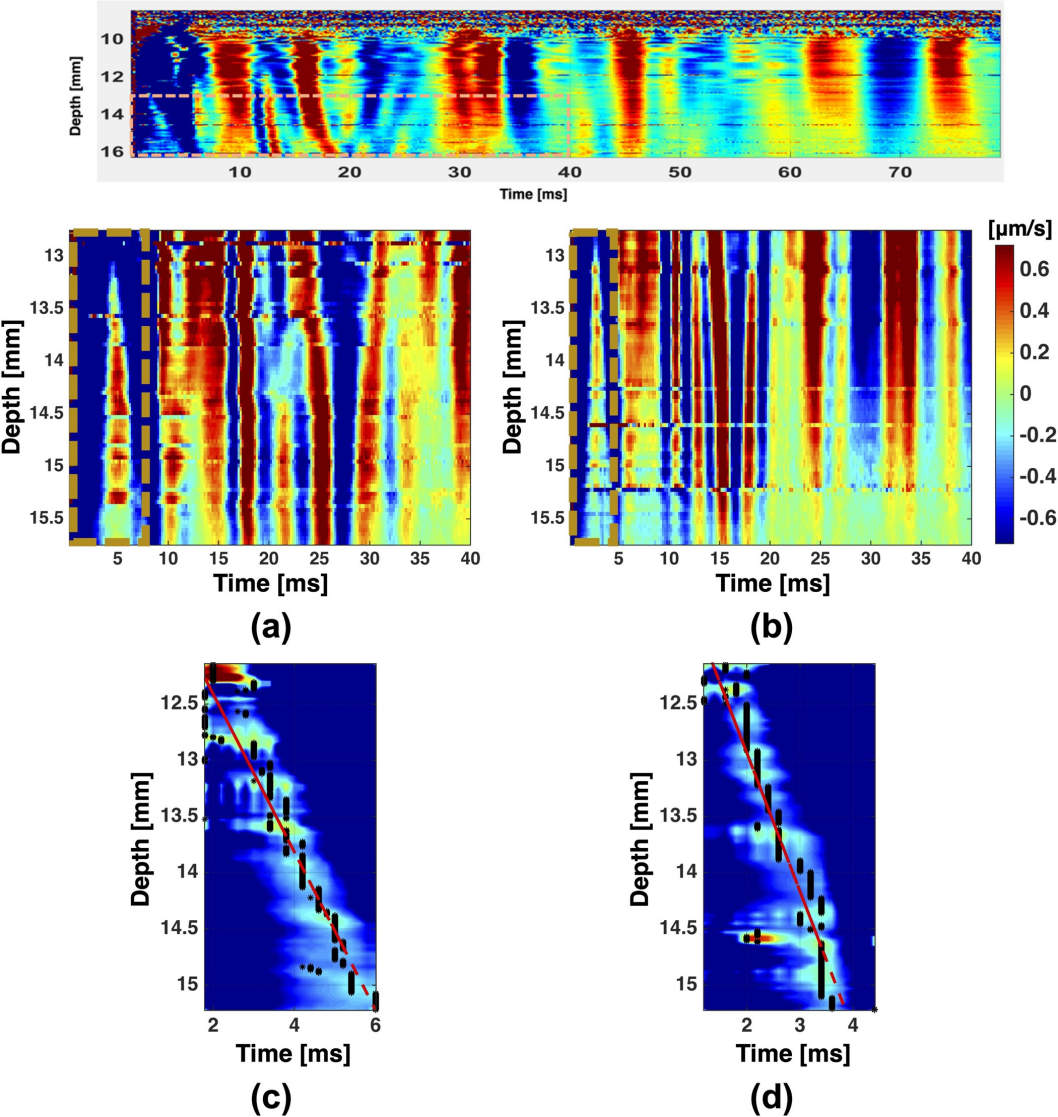
The arrival-time plots cropped around the ultrasound focus for (a) 0.3% agarose and (b) 0.4% agarose homogeneous phantoms. (c) and (d) Directionally filtered axial displacement fields within the brown dashed rectangles indicated on (a) and (b), respectively. The false-color scale indicates the shear wave speed (SWS).

The reflected SW arrival-time plots for two homogeneous phantoms with agarose concentrations of 0.3% and 0.4% are copped around the focal point to remove noises at coupling medium and reverberations in later sampling time, as shown in Fig 3(A) and 3(B). The SW arrival-time plots show the axial velocity fields of the reflected SWs with respect to the recording times. The region indicated by the brown dashed rectangle in each plot is the axial velocity field induced by the SWs reflected from the top and bottom faces of the sample. Hence, a directional filter can be applied to better reveal the SW reflected from the top surface, as shown in Fig 3(C) and 3(D), respectively. Linear regression (shown as the red dashed line) was used to fit the slope of the directionally filtered reflected SW to yield an initial estimate of the SWS of the sample.

**Fig 4:**
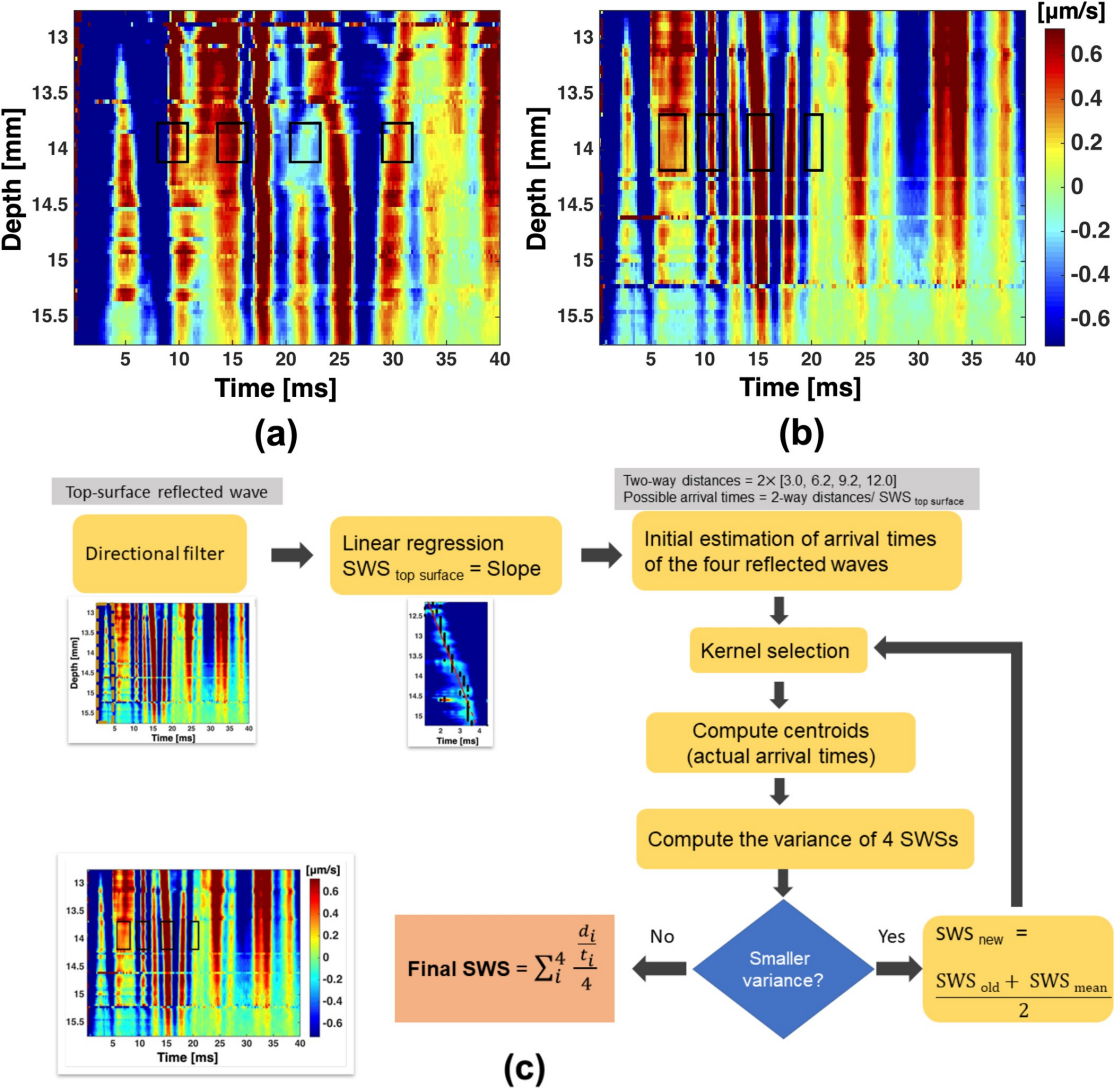
Arrival-time plots for (a) 0.3% and (b) 0.4% homogeneous agarose phantoms, with black rectangles indicating the respective shear waves reflected from the four vertical faces of the sample. (c) Summary flow chart of the iteration process.

The initial estimated SWS was used to estimate the arrival times of the SWs reflected from the four side boundaries. An iterative process was implemented to search for the most-probable SW wavefront closest to the respective estimated arrival time. The arrival times of the selected SW wavefronts were then calculated using the time-to-centroid method, which yielded four SWSs based on the known propagation distances. A second iterative process was implemented to finalize the selection of the four SW wavefronts by minimizing the variance in the four resulting SWSs. The iterative process and outcomes are shown in Fig 4, where the black rectangular boxes indicate the four finally selected SWs. Table 1 summarizes the averaged SWS calculated for four different phantoms with different heights. The accuracy of the measurement is compared with the reference SWS estimated using LSCI-SW.

**Table 1:** Summary of the shear wave speeds (SWSs) estimated for different homogeneous phantoms by the single-element transducer ultrasound shear wave system and the laser-speckle contrast shear wave imaging (LSCI-SW) system.

Phantom composition	Phantom height [mm]	SWS_Ultrasound_ [m/s]	SWS_LSCI-SW_ [m/s]	Deviation [%]
0.3% agarose	4.7	0.81	0.80	1.65
	5.1	0.81		1.78
	5.6	0.78		–2.97
0.4% agarose	4.7	1.15	1.17	–1.74
	5.1	1.19		1.45
	5.6	1.20		2.23
3.5% gelatin	6.2	0.74	0.76	–3.06
5% gelatin	6.5	1.18	1.15	2.41

**Fig 5:**
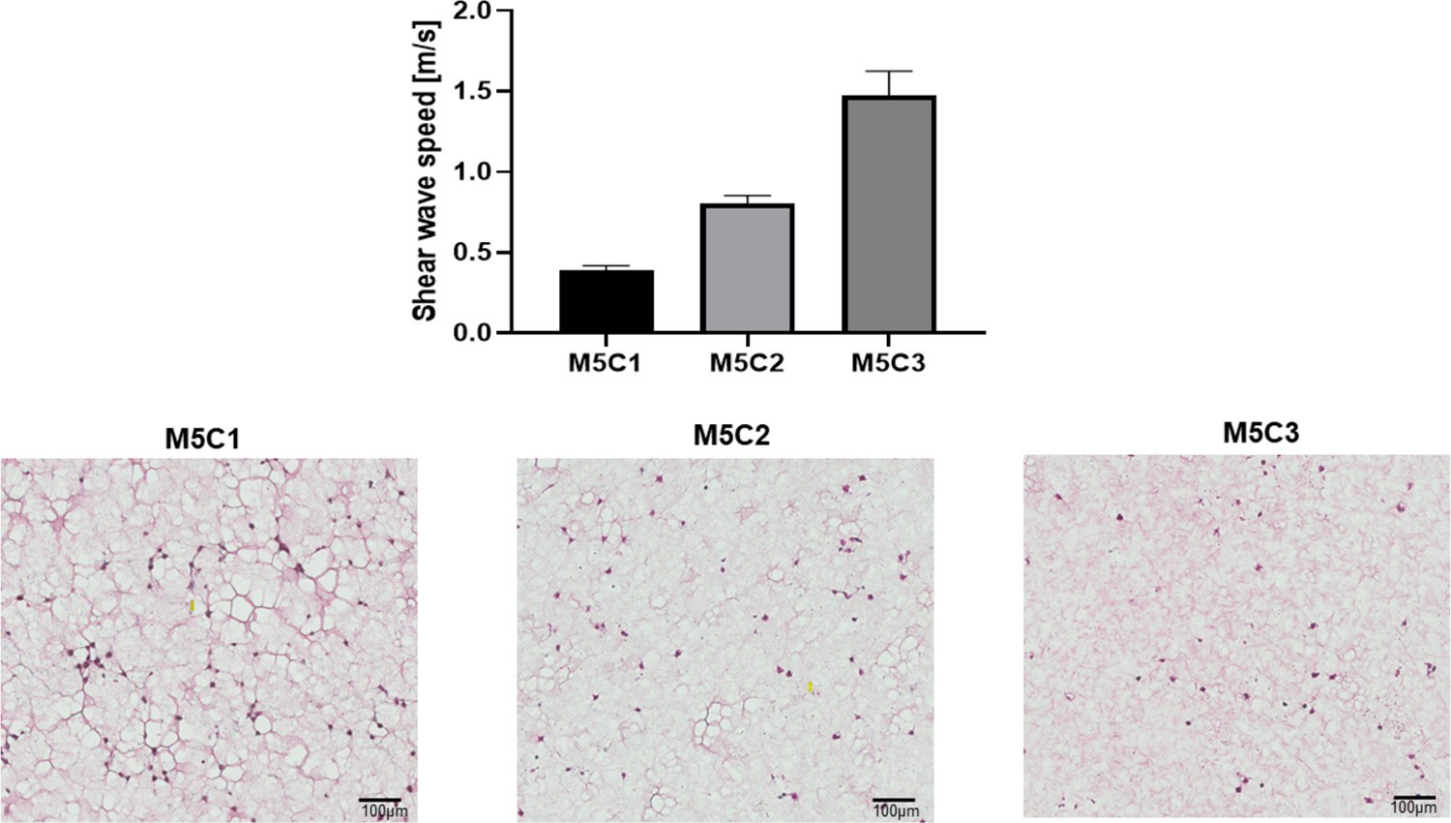
SWSs measured for cell-laden gels with 5 mg/ml Matrigel and 1 mg/ml (M5C1), 2 mg/ml (M5C2), and 3 mg/ml (M5C3) collagen type I. Data are mean and standard-error values for three independent experiments. The lower panels are H&E-stained images of the respective regions of the cell culture samples. The pore size in the matrix decreased as the collagen concentration increased.

Fig 5 demonstrates the SWSs measured for cell-laden gels with different concentrations of collagen type I. The mean SWSs calculated for cell culture samples with 5 mg/ml Matrigel and 1, 2, and 3 mg/ml collagen type I were 0.47, 0.80 and 1.47 m/s, respectively. The SWS increased with the collagen concentration, whereas the pore size in the matrix network decreased with increasing collagen concentration. These findings are consistent with those of a previous study that used two ultrasound transducers for SWEI measurements [29].

**Fig 6:**
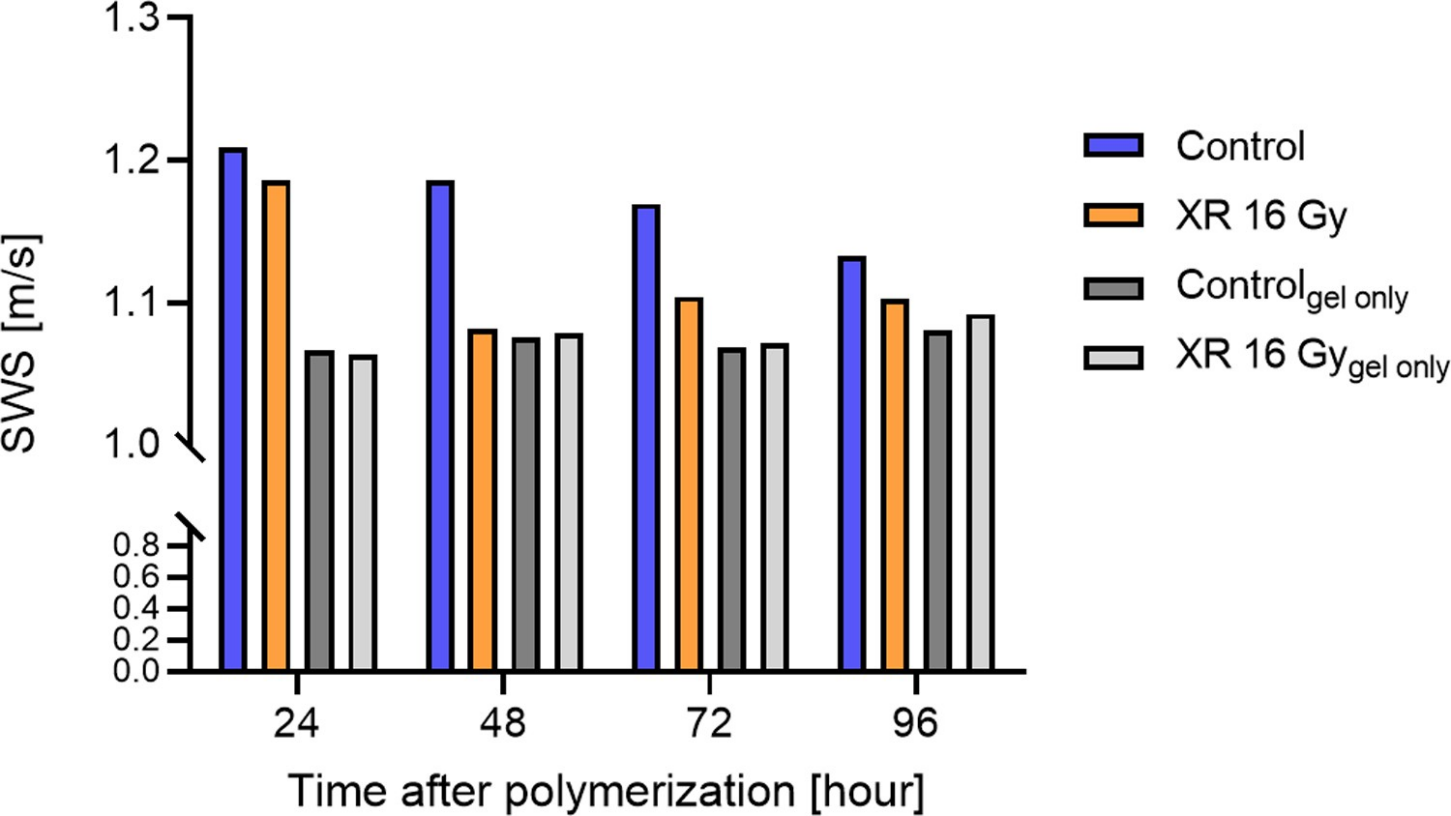
Temporal stiffness dynamics in samples without embedded cells and with cell-laden gels with or without XR at 24 hours.

An attractive aspect of the proposed platform is its ability to provide temporal observations of the elastic properties of a cell-populated biomatrix without damaging the sample. Fig 6 shows an example of the temporal dynamics of the SWSs measured for a matrix comprising 5 mg/ml Matrigel and 3 mg/ml collagen remodeled by the human hepatocellular carcinoma cell line. A gel sample with the same composition but without embedded cells served as be a comparison. The sample was irradiated with 225-kVp ionizing photons at 16 Gy (SmART, Precision X-Ray, Connecticut, United States) at 24 hours after polymerization, and SWEI was performed until 96 hours. While there was a trend for the SWS to decrease in the cell-laden sample during the incubation period, the SWS remained unchanged for the sample without cancer cells regardless of whether or not X-ray irradiation (XR) was applied. In contrast, the application of XR significantly lowered the SWS of the 3D cell culture.

## Discussion

We present an SWEI platform with a single-element transducer that generates SWs and detects the arrival times of reflected waves in a millimeter-sized 3D culture system. The designed fixture ensured that the position of the ultrasound transducer relative to the sample remained constant throughout the measurements. However, a location offset of the ultrasound transducer may still occur during its installation and when mounting the fixture onto the sample container. We therefore averaged the speeds of four reflected waves when determining the stiffness of the sample. We compared the SWSs measured using an LSCI-SW system with those obtained using a single-element transducer ultrasound SW system, and found that the deviations between the two platforms were generally less than 3% for both nonviscous agarose and viscous gelatin samples. Moreover, the height of the phantom had no significant impact on the SWS measurements.

We further investigated the feasibility of measuring the stiffness of cell culture samples. We varied the concentration of collagen to produce 3D cell cultures with different matrix stiffnesses, since [32,33] have reported that a higher concentration of collagen type I increases the scaffold stiffness. In order to distinguish the SW wavefronts reflected from the four side boundaries of each sample, the increments in the one-way distances between the focal point of the ultrasound transducer and the side boundaries were evaluated empirically. For a nonviscous sample with an SWS of 1.3 m/s or less, a minimum one-way distance increment of 3 mm was required for the samples investigated in this study. However, we were still able to discriminate reflected SWs in a viscous biological gel, in which SWs propagate faster than 1.7 m/s. For samples with even higher stiffnesses, cultures with larger dimensions should be considered. On the other hand, a smaller sample will facilitate the diffusion and availability of nutrients and gasses, and so is more suitable for a softer culture matrix. The SW wavelength is determined by the characteristics of the medium and the SWS, and is usually within the range of several hundred micrometers to 1 millimeter in the proposed platform, which will be at the lower end of the culture dimensions. The ratio of the wavelength to the size of cell aggregates should also be taken into consideration. In the present work, the cells were resuspended as a single-cell solution before being embedded into the ECM. By the end of incubation, the cell aggregates or tumorspheres were smaller than 100 μm, which is shorter than the SW wavelength and hence ensured reliable SWEI interrogations.

The proposed platform utilizes a single-element ultrasound transducer and 1D correlation tracking methods to measure shear wave speed, which is similar to the setting of transient elastography [34–36]. The speed of the wave is obtained as the slope of a straight line that is linearly fitted to the shear-wave arrival time versus depth in transient elastography. While we also derive an initial speed estimate by the slope in a plot of arrival time versus depth, the proposed platform is different from transient elastography. First, we use ARF to generate the matrix perturbations inside the sample. In contrast, in transient elastography, the strongest waves arise from the edge of the thumping piston. Those waves converge on the ultrasound axis and, after some distance, travel down the axis at speed close to the shear-wave speed. A displacement “M-mode” recorded by RF A-line is then linearly fitted for the shear-wave arrival time versus depth [34]. In the proposed platform, we calculated the slope based on the reflected shear waves but not the wave right after the ARF push. Finally, the speed of reflected waves from the top surface is utilized to estimate the other four reflected waves side boundaries. Although both the proposed platform and transient elastography have a common assumption on the homogeneous stiffness, we derive the measurement based on the wave propagations along the ultrasound axis and lateral directions.

AFM has been widely adopted to characterize the stiffness of biomaterials by making micro-indentations or nano-indentations on the sample surface (to <10% of sample thickness) [10]. This method provides a spatial resolution of elasticity on the scale at which a single cell feels its matrix [10]. In contrast, the biomaterial in our proposed platform is assumed to have a homogeneous elasticity at the millimeter scale by providing an average SWS in a defined volume. It has been shown that the surface properties could differ from the bulk properties, and measurements made using a smaller indenting tool are less likely to represent the sample from the macroscopic point of view [4]. Assessments of the elasticity of biomaterials should therefore examine bulk samples and their constituents at microscale dimensions, not only because the stiffness measurements themselves are likely to be different at different scales, but also because at these smaller dimensions the cells sense and respond to its mechanical characteristics [37]. Each investigative technique has specific strengths and drawbacks in terms of technical difficulty, sample dimensions, biological relevance, accessibility for imaging, and specific amenability for a given investigation. The system used in a particular situation is generally chosen based on a trade-off between these traits that best suits the specific biological question being addressed.

AFM and other interrogation methods such as Brillouin microscopy and optical coherence elastography [38], which offer micro-cale resolution for mechanical characterization, are generally appropriate for investigations of cellular or subcellular mechanosensing and mechanotransduction. In contrast, the platform proposed in this study measures the stiffness at the macroscale (on the order of millimeters). This makes it more appropriate for revealing how a group of cells remodel their surrounding matrix and how interventions on mechanical properties could affect the embedded cell aggregates as a whole. If spatial heterogeneity of the stiffness within the sample is not of particular interest, the platform using a single-element ultrasound transducer provides a technically noninvasive and straightforward approach for elasticity measurements on optically turbid biomaterials.

While assessing shear elasticity at the millimeter scale, the platform with the noninvasive approach reveals the temporal dynamics of matrix stiffness. Emerging discoveries have suggested that a certain degree of temporal dynamicity resulting from either stress relaxation or stress stiffening can lead to large differences in cellular responses. The scientific community has started shifting its attention to active interventions that involve changing the ECM stiffness during incubation. It has been commonly reported that the speed of cells migration is much slower in 3D than in glass-substrate (2D) environments [39,40]. The speed of 3D migration is approximately half to one-tenth of that measured in 2D conditions [40,41]. There have been studies characterizing cell migration within 3D environments at the time scale of days [42,43]. In the present study, every 24 hours have we demonstrated the dynamics of stiffness through a novel platform. We think it would be an appropriate interval characterizing the stiffness dynamics.

The different dynamics of SWSs in cell-laden gels and gels without embedded cells as revealed in the present study suggest that active matrix remodeling occurs during incubation. Moreover, we observed that XR treatment resulted in a significant decrease in SWSs in cell cultures but not in samples only comprising Matrigel and collagen. These distinct responses to XR confirm that temporal changes in SWSs probably result from interactions between cells and their surrounding matrix.

It has been recently been recognized that XR affects remodeling of the tumor microenvironment, involving inflammation, fibrotic responses, and immune activation [44]. Seminal studies have also disclosed that the ECM stiffness is associated with cancer radiosensitivity via the activation of adhesion-mediated integrin [45–47]. These findings were typically based on static elasticity measurements made by the endpoint probing of ECM-coupled hydrogels. We present a new approach to investigating the temporal dynamics of the XR-induced reactions within a millimeter-scale 3D cell culture. The stiffness of the ECM influences the response of solid tumors to radiotherapy, chemotherapy, and immunotherapy [48]. At the same time, novel drugs targeting stiffness-mediated treatment resistance have shown promising results in preclinical studies [44,48]. The present study has produced a platform for investigating the mechanisms underlying how the ECM influences cancer treatment. Using an even smaller ultrasound transducer with a higher central frequency would make it possible to further reduce the size of the cell-laden biomaterial, thereby facilitating the diffusion of agents of interest into the 3D culture.

There are several limitations to the proposed approach. First, the elasticity of biomaterial is calculated by applying a time-of-flight algorithm to the reflected shear waves from the side boundaries of the sample. Therefore, we could not probe the stiffness of a sample without defined boundaries or in live animals. Moreover, the stiffness was derived from the speed of the shear waves induced inside the in vitro 3D matrix. It has been shown that the thin thickness of the hydrogel phantom would bias the measurement of SWS [49]. As the wavelength of the ARF-generated SW in the matrix appears to be about 2.0 mm, we should keep the distance between the focus and the sample surface larger than 1.0 mm. The depth of the biomaterial hydrogels should not be less than 2 mm. Otherwise, the probed stiffness could be an underestimation. Finally, identifying four reflected SWs from sample boundaries is needed, which limits further reductions of the dimension of the hydrogels with high stiffness.

## Conclusions

In conclusion, SWS measurements can be made using a single-element ultrasound transducer for both SW generation and detection. These nondestructive and noncontact stiffness measurements are beneficial to providing the temporal dynamics of matrix stiffness at the macroscale, especially for delicate and optically turbid biomaterials such as engineered tissue samples.
